# Protocol refinement for a diabetes pragmatic trial using the PRECIS-2 framework

**DOI:** 10.1186/s12913-021-07084-x

**Published:** 2021-10-02

**Authors:** Russell E. Glasgow, Dennis Gurfinkel, Jeanette Waxmonsky, Jenny Rementer, Natalie D. Ritchie, Jennifer Dailey-Vail, Patrick Hosokawa, L. Miriam Dickinson, Bethany M. Kwan

**Affiliations:** 1 University of Colorado Anschutz Medical Campus, 13199 E Montview Blvd Ste 210, Aurora, CO 80045 USA; 2 VA Eastern Colorado QUERI and Geriatric Research Centers, 1055 Clermont St, Denver, CO 80220 USA; 3 Denver Health and Hospital Authority, 777 Bannock St, Denver, CO 80204 USA

**Keywords:** Type 2 diabetes, Shared medical appointments, Design, Pragmatic research, PRECIS-2, Planning, Implementation science, Transparency

## Abstract

**Background:**

This report describes how we refined a protocol for a pragmatic comparative effectiveness study of two models of an evidence-based diabetes shared medical appointment intervention and used the PRECIS-2 rating system to evaluate these adaptations.

**Methods:**

We report primary data collected between June and August 2019, and protocol refinements completed between 2018 and 2020. Twenty-two members of the study team collaborated in protocol refinement and completed the PRECIS-2 ratings of study pragmatism. We discuss study design refinements made to achieve the desired level of pragmatism vs. experimental control for each of the nine PRECIS-2 dimensions. Study team members received training on PRECIS-2 scoring and were asked to rate the study protocol on the nine PRECIS-2 dimensions. Ratings were compared using descriptive statistics.

**Results:**

In general, the PRECIS-2 ratings revealed high levels of pragmatism, but somewhat less pragmatic ratings on the categories of Delivery and Organization (costs and resources). This variation was purposeful, and we provide the rationale for and steps taken to obtain the targeted level of pragmatism on each PRECIS-2 dimension, as well as detail design changes made to a) make the design more pragmatic and b) address COVID-19 issues. There was general agreement among team members and across different types of stakeholders on PRECIS-2 ratings.

**Conclusions:**

We discuss lessons learned from use of PRECIS-2 and experiences in refining the study to be maximally pragmatic on some dimensions and less so on other dimensions. This paper expands on prior research by describing actions to achieve higher levels of pragmatism and revise our protocol fit to the changed context. We make recommendations for future use of PRECIS-2 to help address changing context and other strategies for the planning of and transparent reporting on pragmatic research and comparative effectiveness research.

**Trial registration:**

Clinicaltrials.gov Registration ID: NCT03590041.

**Supplementary Information:**

The online version contains supplementary material available at 10.1186/s12913-021-07084-x.

## Introduction

Failure to replicate research results in real world settings [1, 2] has received increased attention [3, 4], and demands proactive methodologies rather than “business as usual” to improve health services research [5, 6]. Although a multifaceted issue, part of ‘failures to replicate’ may not be actual failures, but rather reflect that interventions found to be efficacious under one set of conditions do not generalize to other settings, conditions and populations [4, 7]. One step for addressing replication concerns is to enhance transparency in reporting on context, including the extent to which study features mirror or contrast with aspects of usual care. Although participating patient details are now addressed by CONSORT [8–10] and other reporting standards, other contextual factors such as selection, exclusions, participation and representativeness at the broader levels of settings (communities, healthcare systems, clinics) and staff are often unreported [4]. Fortunately, methods for addressing these generalizability issues are emerging, including the NIH Reporting requirement [3], the Standard Reporting for Implementation Science (StaRI) guidelines [11] and the updated Pragmatic-Explanatory Continuum Indicator Summary-2 (PRECIS-2) and the just published PRECIS-2 Provider Strategies (PRECIS-2-PS) systems [12–14].

In contrast to more traditional randomized explanatory or efficacy trials, pragmatic trials are typically characterized by few exclusions for settings, staff and patients, using practical measures of outcomes important to patients and decision makers, and comparing real world intervention alternatives [15]. Pragmatic trials having the features above evolved as a way to address the well-documented gap between standard RCT research findings and practice [16, 17]. In particular, the PRECIS-2 rating system [12] is widely recommended to plan pragmatic trials [18–21]. As demonstrated in earlier publications and below, it is also possible to use the PRECIS tools to report on changes in design during a trial or to categorize published trial designs [18, 22]. To aid trial design decisions, PRECIS-2 is represented as a 9-spoked ‘wheel’ with the following domains: 1) eligibility criteria (who is selected to participate in the trial?); 2) recruitment (how are participants recruited into the trial?); 3) setting (where is the trial being done?); 4) organization (what expertise and resources are needed to deliver the intervention?); 5) flexibility delivery (how is the intervention delivered?); 6) flexibility adherence (what measures are in place to make sure participants adhere to the intervention?); 7) follow up (how closely are participants followed-up?); 8) primary outcome (how relevant it is to participants?); and 9) primary analysis (to what extent are all data included?).

PRECIS-2 may best be used in partnership with stakeholders as part of the study design process [20]. Yet even when trials are planned in partnership with stakeholders, grant proposals are hypothetical in nature and intended to demonstrate feasibility, rigor, and importance to reviewers. Given the time lag between when a study is proposed, actually funded and the trial actually begins, even the best planning cannot predict the exact context, staff, resources, and motivations present at the time a trial begins. To ensure ongoing fit to changing context while retaining rigor, pragmatic trials should anticipate the need for protocol refinement when launching (and potentially at key points during the study) to fully specify the trial procedures, measures, materials, data sources, timelines, and participant and setting eligibility criteria. Any *changes* to the originally approved or agreed upon methods or study design elements should be guided by a systematic process and framework such as the PRECIS-2, well-documented, and communicated transparency. For pragmatic trials, protocol refinements made during the trial are usually driven by contextual changes. For example, a change in the electronic health record software used by a participating healthcare organization could necessitate a change in how clinical outcomes are operationalized and assessed.

Many reports detail use of PRECIS-2 for planning (www.precis-2.org) [19], including for reviewing and reporting findings [18, 22], yet to our knowledge only one has focused on protocol adaptations or design changes and that one only briefly [23]. This report describes how we refined a protocol for Invested in Diabetes, summarized below, which was intended as a pragmatic comparative effectiveness study of two models of evidence-based diabetes shared medical appointment (SMAs). The “main objective” for this study explicitly included protocol refinement. We previously reported on pre-implementation adaptations to the *comparator interventions* to fit context (i.e., planned adaptations to intervention content, packaging, delivery, as well as training for those delivering the interventions) as a step in the Replicating Effective Programs framework used to guide the study [24], yet other aspects of protocol refinement have not yet been reported or viewed through a PRECIS-2 lens. In 2020, the Invested in Diabetes study - at about the midpoint of patient enrollment - was also faced with responding to the COVID-19 pandemic, requiring further protocol refinements to both the intervention and the research design. We used PRECIS-2 as a lens for describing and designing the process of protocol *refinements* to be more pragmatic and feasible within the context of COVID.

The purposes of this paper are to describe how we: 1) refined our study protocol using a participatory team science approach [25, 26] and the PRECIS-2 framework; 2) responded to the changing context in implementation settings and due to COVID-19; 3) identified similarities and differences in perceptions of study pragmatism among different types of raters; and 4) to discuss lessons learned and recommendations for future use the PRECIS-2 system and related strategies to aid protocol refinement.

## Intervention approach and methods

### Design

The Invested in Diabetes study is a cluster randomized pragmatic trial currently underway in 22 primary care practices in Colorado and Kansas. Its overall objective is to compare the effectiveness of patient-driven vs. standardized diabetes shared medical appointments (SMAs) on patient-centered outcomes, especially diabetes distress. Standardized SMAs were group visits led by a health educator with a set order of session topics. Patient-driven SMAs were delivered collaboratively by a multidisciplinary care team consisting of a health educator, behavioral health provider, and a peer mentor, and patients choose the topic order.

During the pre-implementation phase for this project, the research team engaged practice stakeholders and refined the study protocol as originally designed to better align with real world systems and processes of care (i.e., to make the protocol more pragmatic). The PRECIS-2 was used to structure this process of protocol refinement.

### Setting

Our goal was to study diabetes SMAs across a variety of primary care settings (e.g., federally qualified health centers (FQHCs), internal and family medicine practices serving patients with commercial insurance, and community mental health centers) who had integrated behavioral health providers. Community mental health centers were ultimately unable to participate due to Colorado Medicaid reimbursement changes that impacted their ability to provide health education services. The final randomized study cohort included 12 FQHCs and 12 primary care practices from practice-based research networks [24].

### Original study protocol

Invested in Diabetes used cluster randomization to assign practices to either patient-driven or standardized diabetes SMAs. In both SMA conditions, the curriculum used is Targeted Training in Illness Management (TTIM), which has been shown to be effective for patients with diabetes and serious mental illness [27, 28]. Existing practice staff deliver the assigned SMAs using study-provided TTIM materials. Eligible primary care practices must be one of the practice types above, have a roster of ≥150 patients with type 2 diabetes, and have integrated behavioral providers to serve on the multidisciplinary care team. Patient eligibility criteria are broad: patients must be ≥18 years of age, have type 2 diabetes, not pregnant or planning on becoming pregnant, and not be in hospice or within 6 months of the end of life. A mixed-methods evaluation includes quantitative (practice- and patient-level data) and qualitative (practice and patient interviews, observation) components. The primary patient-centered outcome, selected by patient stakeholders, is diabetes distress [29, 30]. Secondary outcomes include autonomy support, quality of life, and diabetes self-management behaviors, clinical outcomes, patient reach and engagement, and practice-level value and sustainability. The primary source of clinical data is from each practice’s electronic health records, using data collected from routine care. Further details on the study protocol have been previously published [31].

### Stakeholder engagement in protocol refinement

During the pre-implementation phase (study year 1), we engaged clinic and patient stakeholders to refine the study protocol and address potential barriers to implementation of the protocol. Stakeholders included two members of practice leadership, two diabetes educators or nutritionists, one clinician, and five patient stakeholder who had long-standing type 2 diabetes or were family members of patients with diabetes. The study team discussed elements of the study protocol with interested practices, including those who ultimately enrolled in the study and those who did not enroll. Protocol elements discussed included practice and patient eligibility criteria, research data collection plans at the both the patient and practice level, and expectations for clinical sites to use usual practices and personnel for patient recruitment, delivery of care, and documentation and billing. These discussions occurred in a variety of forums, including between the investigators and practice leadership and clinical teams during recruitment and initial project start-up meetings, between practice facilitators and clinical teams during trainings and coaching sessions, and between investigators and practice and patient stakeholder representatives during research team and stakeholder engagement meetings.

### Protocol refinement and operationalization

Based on stakeholder input, the research team refined the study protocol to increase practice willingness to participate, and ensure the protocol would reflect real-world practices and resources. The practice facilitators and investigators iteratively discussed potential changes with practice representatives until reaching satisfactory decisions. In refining the protocol, there were aspects of the study design that were ultimately not pragmatic for the practices (e.g., patient-reported outcomes measures). In Table 3, we summarize the protocol refinements by PRECIS-2 domain and describe how the study team, patient stakeholders, and participating practices have operationalized the protocol to fit their usual processes of care. In addition to the protocol refinements made in the pre-implementation phase in 2018, we also describe protocol refinements made during the implementation phase in response to the COVID-19 pandemic in 2020.

### Pragmatic design ratings using the PRECIS-2

[23, 32] Approximately 9 months into implementation of the study, The study team reflected upon the extent to which aspects of the original study protocol were more or less pragmatic using the PRECIS-2. PRECIS-2 ratings were completed by 21 individuals, including 6 clinician investigators, 6 non-clinician investigators, 6 research staff, and 3 practice-interfacing staff. The research team was asked to rate the original Invested in Diabetes research protocol according to how pragmatic it was on each of the PRECIS-2 domains. Following a one-hour presentation by the first and senior authors on PRECIS-2, including examples of common issues in rating PRECIS-2 dimensions and questions and answers, research team members (with the exception of patient representatives) were given relevant excerpts from the funded study proposal and a spreadsheet defining each of the PRECIS-2 domains and rating criteria. They independently rated each domain on a scale of 1 (very explanatory) to 5 (very pragmatic) based on their own perspective of the protocol’s consistency with PRECIS-2 definitions with reference to usual care systems and processes. Ratings were conducted between June and August 2019. We report ratings by different subgroups, but efforts were not made to resolve discrepancies.

### Analyses

We used descriptive statistics to summarize mean ratings for each domain overall and by rater type and plotted the mean responses for each domain on the PRECIS-2 spider plot. To address our third study aim, we compared ratings by those who worked directly with the practices to ratings by research team members who were not directly engaged with practices. Our hypothesis was that those who worked directly with practices would rate the protocol as less pragmatic.

## Results

### Participating practice characteristics

Table 1 summarizes the characteristics of participating practices as previously described [24]. As can be seen, there was a diversity of practice types and sizes, and many had a substantial minority or uninsured population.

**Table 1:** Practice Characteristics, Colorado and Kansas, 2018–2021

Contextual Characteristics	Overall (***n*** = 22)	Patient-Driven Condition (***n*** = 11)	Standardized Condition (***n*** = 11)	Difference across conditions*
Practice Type: N (%) Federally Qualified Health Centers	12 (54%)	5 (45%)	5 (45%)	*p* = 1.00
Practice Location: N (%)				
Urban	17 (77%)	9 (82%)	8 (73%)	*p* = 1.00
Rural	3 (14%)	1 (9%)	2 (18%)	
Suburban	2 (9%)	1 (9%)	1 (9%)	
Estimated No. of Diabetes Patients: median (range)	549 (90–4000)	500 (90–4000)	576 (214–2112)	*p* = 0.48
Hispanic/Latino Patients > 10%: N (%) of practices	10 (67%)	5 (63%)	5 (71%)	*p* = 1.00
Minority Patients > 20%: N (%) of practices	13 (87%)	5 (71%)	8 (100%)	*p* = 0.20
Payer Mix: Median (range)				
Private insurance fee for service (FFS) or preferred provider organization	11 (3–70)	12 (3–64)	10 (5–70)	
Private managed care	11 (5–35)	15 (10–20)	10 (5–35)	
Medicare	19 (2–60)	20 (9–30)	14 (2–60)	
Medicaid	40 (2–63)	30 (5–60)	55 (2–63)	
Other public insurance	3 (0–5)	3 (0–5)	3 (0–5)	
Self-pay or uninsured	10 (0–92)	6 (0–92)	10 (3–36)	
Unknown	0 (0–10)	0 (0–10)	0 (0–5)	
Other	4 (0–100)	0 (0–0)	9 (0–100)	
Private (FFS + Managed Care) > (Medicare + Medicaid + Other Pub)	3 (19%)	2 (29%)	1 (11%)	*p* = 0.55
No. Clinicians with prescribing privileges: Median (range)	8 (2–65)	7 (2–39)	8 (3–65)	*p* = 0.39
No. Staff eligible to be health educator: median (range)	2 (1–6)	2 (1–6)	3 (1–6)	*p* = 0.79
Previous experience with SMAs: N (%)	10 (45%)	5 (45%)	5 (45%)	*p* = 1.00

### PRECIS-2 ratings

**Fig. 1:**
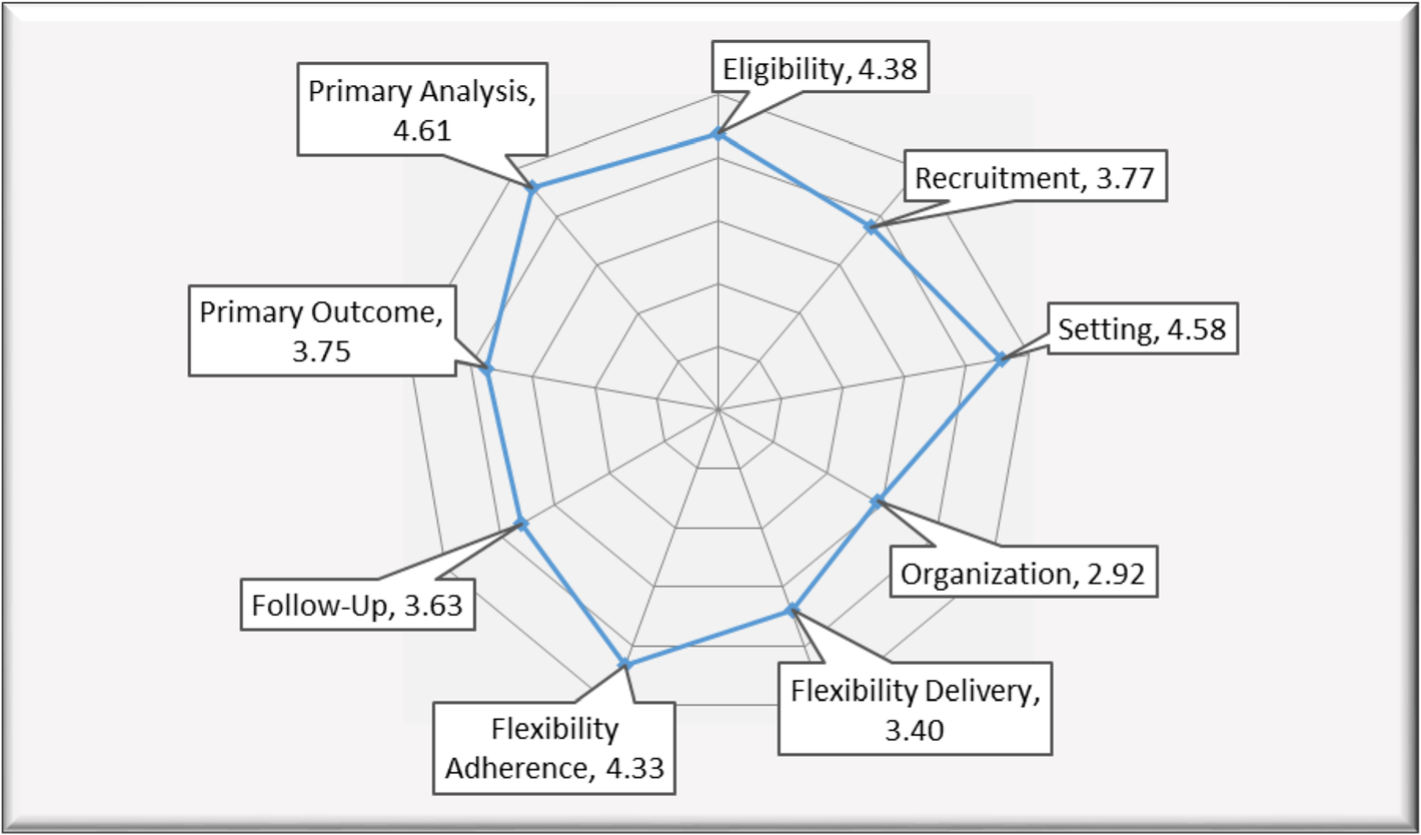
PRECIS-2 ratings of the study protocol by Invested in Diabetes study team. Radar plot showing average study protocol ratings by study team on the nine PRECIS-2 domains, with points closer to center representing explanatory ratings (1) and points closer to the edge representing pragmatic ratings (5). Legend: 1 = very explanatory. 5 = very pragmatic

Figure 1 shows the spider plot for overall study team PRECIS-2 domain ratings for the originally proposed study protocol. Overall, the average rating for all domains was 2.92 or higher, showing that the study team generally perceived the study to be pragmatic. The most pragmatic domains (the most patient-centered and consistent with usual processes of care) included: 1. Primary analysis (intent-to-treat analysis using all available survey and electronic health records data); 2. Setting (primary care practices with ≥150 adult patients with type 2 diabetes and existing integrated behavioral health); 3. Participant eligibility (adults with type 2 diabetes, with the exception of those pregnant, with cognitive impairment, or with limited life expectancy); and 4. Flexibility - Adherence (practices use standard processes such as reminder calls to encourage patient attendance). These were all rated with average ratings exceeding 4.33.

The PRECIS-2 domains of 5. Recruitment (practices invite patients to participate in SMAs as part of their regular health care, using existing resources and processes of care); 6. Primary outcome (diabetes distress, selected by the project’s patient stakeholder partners); 7. Follow-up (patient-reported data collected at baseline and follow-up as part of SMAs; clinical outcomes are secondary use from the EHR); and 8. Flexibility - delivery (core components of SMA delivery established by protocol with required use of the TTIM curriculum) were rated moderately pragmatic, with average PRECIS-2 ratings between 3.40 and 3.77. Finally, 9. Organization (health education, behavioral health, scheduling resources available plus physical space to hold groups) was consistently rated as the least pragmatic dimension (average score of 2.92), possibly because of the low level of resources for conducting research in the participating settings.

Table 2 shows domain ratings by different rater types. There was generally high agreement on the PRECIS-2 ratings, especially on Flexibility-Delivery, Organization, Follow-up, Outcomes and Analyses, with differences among rater groups of .5 or less on mean ratings for these dimensions. The largest differences among types of raters were on the dimensions of Eligibility, Setting, Recruitment and Flexibility-Adherence, with researchers rating the design as more pragmatic than other groups. All rater types agreed that the organization dimension was the least pragmatic.

**Table 2:** Mean PRECIS-2 domain ratings for Invested in Diabetes study protocol by study team member rater type

	Eligibility	Recruitment	Setting	Organization	Delivery	Adherence	Follow Up	Outcomes	Analysis
Clinician investigator (*n* = 6)	4.67	3.67	4.5	2.83	3.33	4.17	3.33	3.83	4.67
Non-clinician investigator (*n* = 6)	4.50	4.33	4.33	3.08	3.17	4.83	3.75	3.50	4.67
Research staff (*n* = 6)	4.00	3.75	4.50	2.75	3.42	4.33	3.75	4.00	4.46
Practice-interfacing staff (*n* = 3)	4.33	3.33	5.00	3.00	3.67	4.00	3.67	3.67	4.67
Total investigators (*n* = 12)	4.58	4.00	4.42	2.96	3.25	4.50	3.54	3.67	4.67
Total non-investigators (*n* = 9)	4.17	3.54	4.75	2.86	3.54	4.17	3.71	3.83	4.56
TOTAL (*n* = 21)	4.38	3.77	4.58	2.92	3.40	4.33	3.63	3.75	4.61

### Protocol refinement to fit usual processes and systems of care

During the protocol refinement period, the study team, clinicians and delivery staff, and practice representatives operationalized and/or refined the study protocol to best fit the usual processes and systems of care while still fulfilling study objectives. Table 3 presents the protocol refinements by PRECIS-2 domains and accompanying rationale. Without inherently changing the protocol, we specified and/or adopted detailed procedures and materials for the outcomes and follow-up data collection and procedures (EHR data extracts; patient reported outcome (PRO) measures, scoring and tracking; attendance; fidelity; cost collection; and patient and practice interviews).

**Table 3:** Invested in Diabetes protocol refinements by PRECIS-2 domain, Invested in Diabetes study team, 2018–2021

PRECIS-2 domain	Original protocol	Protocol refinement	Reason for refinement
Eligibility	• Patients are adults with type 2 diabetes who are existing patients of the practice; excludes pregnancy or plans to become pregnant within 6 months, life expectancy less than 6 months, cognitive inability to participate, plans to leave area within next year.	• Practices encouraged to recruit any adult with type 2 diabetes they believed would be able to participate in and benefit from SMAs (i.e., no explicit assessment of eligibility criteria; providers review lists of patients to assess suitability) • Pregnancy exclusion criterion would be applied analytically based on EHR data.	• Not typical for practices to ask patients if planning to become pregnant or leave the area prior to offering care; • Assessment of cognitive ability, life expectancy, and other “suitability” factors based on provider judgment
Recruitment	• Practices would recruit patients using existing personnel and processes of care	• Practices shared strategies used to meet recruitment goals during practice stakeholder calls. • Engaged patient stakeholders helped develop marketing materials. • Research team provided recruitment fliers and coaching sessions to support recruitment strategies.	• Recruitment was listed as a top barrier from every practice and they needed extra support and guidance to achieve recruitment goals.
Setting	• Diverse clinic settings: FQHCs, private practices, and community mental health centers with integrated behavioral health and primary care, including small/large and urban/suburban/rural sites in Colorado. • Practices had ≥100 adult patients with type 2 diabetes (able to commit to 72 patients over 2 years, including 60 with complete PRO data)	• No community mental health centers recruited • Allowed “half-sites” to provide 36 patients instead of 72 • Allowed participating organizations to combine smaller practices into one “site” for randomization purposes	• Policy-level changes to billing/payment structures for community mental health centers came into effect during practice recruitment. • Smaller practices allowed to participate to reach practice recruitment goals and to ensure results were relevant to a greater range of practice sizes
Organization	• Existing personnel required prescribing providers (MDs/DOs), integrated behavioral health providers (master’s and doctoral level), health educators (nurse, health educators or certified diabetes educators), and SMA coordinators. • Patient-driven practices required to identify existing patients with type 2 diabetes to serve as peer mentors • SMAs paid for through billing for prescribing provider office visits.	• Prescribing providers could include PharmDs • Health educators could include Medical Assistants or Community Health Workers, if properly supervised	• PharmDs are also able to provide medication management and can bill for services • Not all practices had formally trained health educators on staff • Not all practices required prescribing provider visits to pay for services
Flexibility-Delivery	• Practices randomized to patient driven or standardized SMA condition and follow delivery core components according to assigned arm • Must use the TTIM curriculum with no additional content • Medical provider addresses medical management needs in group session for 20 min • Once a week for 12 weeks for 60 min sessions in person. • 8–10 patients per cohort	• 6 session model (each 90 min to 2 h in length) • Health educator may also include trained lay personnel (e.g., community health workers, medical assistants) • Medical provider may also include pharmacists • Prescribing provider may meet individually with patients for 5–10 min at some or all sessions • Practices enrolled later in the study may reach fewer patients	• Less burdensome for practices to deliver a 6 session model, which may also support retention • To accommodate staffing demands, availability, and need for Spanish-speaking personnel • Some practices prefer pharmacists meet with patients for diabetes management needs, given large role of medication in diabetes care • Individual visits with medical provider supports higher reimbursement rates toward costs of delivering diabetes SMAs • Practices enrolled later in the study have less time to reach initial goals for recruitment
Delivery-Adherence	• Practice facilitation and observation support fidelity	• Established core vs. flexible components of diabetes SMAs to gauge fidelity • Homework: Leave it up to the practice if they assign homework.	• Practices have different priorities and resources for delivering diabetes SMAs
Follow-up	• PROs required at start and end • Retention efforts conducted as in usual clinical care (e.g., reminder and follow up calls) • Participation included as an outcome (differences in conditions for reach and engagement)	• To get follow-up PROs, practices can call to get these data from patients who do not attend the final SMA session • For patient-driven diabetes SMAs, peer mentors may assist with encouraging patients to attend all SMA sessions • Practices not required to track patients who refused invitation to SMAs	• Difficulties in completing PROs at sessions due to limited time or patient no-show • Overly burdensome for practices to track who they invited to SMAs (they might have a master list of who they contacted and invited - most have a spreadsheet they use to track who they’ve contacted but we do not ask them to report this data)
Outcomes	• 17-item Diabetes Distress Scale (DDS-17); 6-item Health Care Climate Questionnaire (HCCQ); 5-item EQ-5D; 11-item Summary of Diabetes Self-Care Activities (SDSCA); 14-item self-report measure in the Health LiTT tool (53 total survey items) • Surveys administered at first and last sessions • Select items from the Medical Expenditure Panel Survey, National Health Survey, and American Time Use Survey for select patients to assess patient out-of-pocket cost	• PROs for assessment during sessions now limited to 45 survey items • Added Perceived Confidence Scale • 3-item Brief Health Literacy screener instead of Health LiTT • Removed EQ-5D • Baseline surveys administered during first session attended (i.e., the second session for a patient who missed the first sessions) • Follow up surveys administered by phone or mail for patients who did not attend the last session • PROs collected using online survey platforms • Out-of-pocket cost questionnaire assessed among select patients verbally during patient interviews	• Need to limit burden to patients in completing surveys, while minimizing time needed to measure outcomes during sessions • Reprioritizing measures of value and importance to stakeholders • Need to collect baseline surveys for patients who miss the first session; • Need to collect follow up surveys for patients who stop attending • Surveys previously collected on paper were no longer feasible with vSMAs • Reduced burden for practice to recruit for non-PRO surveys; reduced burden for patient to provide responses to study team
Analysis	• Intent to treat analysis • 10 practices per arm (20 total) • 72 patients enrolled per practice (720 per arm)	• Increased number of sites to 22 • Reduced patient enrollment requirements to 36	• Additional sites needed to accommodate half-sites and retain patient recruitment goals (e.g., two half-sites equal 1 full site)

#### Electronic health records (EHR) extracts

In partnership with three participating sites (two with a more developed data and reporting infrastructure and one with a less developed system), we iteratively developed detailed specifications for EHR data extraction (Additional file 1). We created an easy to use protocol and template/mock report tables based on practice input. We made some data elements optional (e.g., foot and eye exams), based on capability of the local EHR (refining our “must haves”). The optional data elements were those that would only be needed to provide sites with feedback reports on the degree to which SMAs influenced their quality metrics, and not our ability to test a priori study hypotheses. Data extraction instructions addressed the selection criteria for patients to include, the time frame for the data pull, and specifics from four data categories: demographics, medications, vital signs and laboratory results, and patient visits. The extraction guide noted details such as the number of diagnosis codes to include, the types of labs to include, and how to report patient insurance status (e.g., patient level vs. visit level). For all sites, we were willing to accept more data than requested and selected only those elements needed.

#### Fidelity observations

The study protocol called for observation of 10% of SMAs to assess fidelity to core components (use of the TTIM curriculum and delivery according to assigned study arm) and the conceptual model (described in more detail elsewhere) [31]. We ultimately decided it was feasible to observe 5–8% of visits or one session per practice per quarter for 6 quarters (6 sessions per practice). In the abstract, one might think that the initial goal of observing 10% of sessions was pragmatic, but even this proved challenging in these settings. We adapted a fidelity checklist (Additional file 2) from previous studies using the TTIM curriculum to capture facilitator role, timing, curriculum content, group engagement, and facilitation style. Seven study team members were trained in a two-hour training, then debriefed after completing their first observation.

#### Patient-reported outcomes (PRO) measures and scoring

The patient stakeholders and investigators reviewed the planned PRO measures from the original proposal, considering the extent to which proposed measures were clinically relevant and actionable as part of the intervention for either patients or the health care team. Guidance from the literature on pragmatic trial design and specifically from our Patient-Centered Outcomes Research Institute funder [15] recommends that PRO measures should not be for research purposes only. As a result, the study team eliminated the originally planned quality of life measure as its clinical utility was unclear. However, we added the Perceived Confidence Scale [33], which more directly matches with the skills-building nature of the curriculum and was endorsed as important by patient stakeholders. We designed an easy to understand patient survey document and scoring instructions for practices, including how to handle missing data.

#### Attendance and PRO tracking

As a pragmatic trial, the majority of measures were designed to be administered as part of clinical care by regular practice staff rather than research personnel. We developed a spreadsheet for practices to capture both session data (presenter, date, provider who was available, TTIM content covered or missing, added content, and peer mentor if applicable) and patient data (attendance, PRO scores). To reduce burden of data collection, practices were instructed to submit the tracking sheet quarterly, but were asked to report monthly attendance via email to fulfill sponsor obligations.

In addition to fully operationalizing the data collection processes, there was considerable work for practices to determine how to operationalize patient recruitment. Practices found different ways to recruit, such as: “warm hand-offs” from providers; systematically reviewing lists of patients with suboptimal diabetes management; using social media to advertise; word of mouth, particularly for practices with peer mentors; and advertising around the clinic using fliers, handouts, and on their waiting room TVs. It was an important aspect of the protocol that practices use regular systems (e.g., registries) and processes (e.g., provider referrals, direct calls to patients) to invite patients to participate in groups as part of their regular diabetes care – rather than for a research study. As a result, much of the patient-level data collection procedures needed to reflect secondary use, such that the data were primarily for clinical or operations use. This is a common approach for cluster randomized trials, and a waiver of patient consent was approved by the Colorado Multiple Institutional Review Board.

##### Dynamic changes in the context of COVID-19

The COVID-19 pandemic significantly impacted the delivery of primary care services. We aimed to be extremely flexible with regard to changes in practice delivery of the SMAs in response to COVID-19, without compromising the integrity of the research. A number of practices who had telehealth capabilities switched to virtual delivery of SMAs (vSMAs). The main aspects which the practices had license to modify delivery was to adopt vSMAs and electronic collection of PROs via online surveys.

Adaptations to the delivery of SMAs for a virtual environment included an introduction to the virtual environment for patients, new “ground rules” required for the online environment, and resources to help with problem-solving technical difficulties. Additionally, practices adapted TTIM materials to better fit the online format, e.g., practices increased use of graphics and images and made materials less text based. Other adaptations included sending materials ahead of time by email or mail to SMA participants, scheduling sessions for shorter amounts of time and smaller cohorts, scheduling virtual prescribing provider visits separately rather than during the vSMA session, and moving patient survey data collection to virtual formats or by phone.

The process of transitioning to vSMAs and electronic PROs was captured in field notes by practice facilitators. As of the writing of this paper, twelve practices had adopted vSMAs, while others continued in-person (depending on local infection rates) or postponed SMAs during the pandemic.

## Conclusions

We found it feasible to utilize the PRECIS-2 system to characterize adaptations to our comparative effectiveness trial. With some exceptions, a wide variety of stakeholders were able to understand and make ratings on the PRECIS-2 domains, with generally high agreement across types of raters and good differentiation between PRECIS-2 scores on those domains intended to be very pragmatic vs. those not. Based on these results, we recommend further use of PRECIS-2 and also the newer PRECIS-2-PS system and below we discuss lessons learned as well as recommendations for future research.

Invested in Diabetes was a stakeholder-engaged trial with an overarching goal to optimize pragmatism across the PRECIS-2 factors. The intent was to design a pragmatic study so that the results would be broadly applicable and thus support SMA (and vSMA) adoption, implementation, and sustainability across diverse primary care settings and patient populations. An iterative, multi-stakeholder team approach was used for protocol refinement and operationalization during the first year of the study. We subsequently applied the same protocol refinement process in response to the COVID-19 pandemic. The protocol refinement and operationalization process was then reflected upon, organized, and reported using PRECIS-2.

### Importance of protocol refinements to ensure ongoing practice engagement

Protocol refinements were designed to align make the study more feasible to conduct in busy and low-resource practice settings. In particular, as the majority of data collection was conducted by regular practice staff, we needed to ensure the evaluation was the least burdensome possible. Practice staff needed more assistance than originally anticipated with regards to recruitment, and needed considerable flexibility in response to COVID-19. This process also helped to retain practice participation in the study. As a cluster randomized trial in which practices are the primary unit of analysis and the source of statistical power, it is critical to prioritize practice needs and prevent their withdrawal.

### Value of the PRECIS-2 framework as a tool for collaborative team science

PRECIS-2 helped the team to collaboratively and transparently develop a shared ‘mental model’ of what we were trying to accomplish. It helped to facilitate understanding among research-focused investigators (who prioritize trial rigor) and the practice-focused implementation team (who prioritize practice relevance). The process of a brief initial introductory ‘training’ session on PRECIS-2, followed by independent ratings of PRECIS-2 dimensions, and then coming together to reflect on results was feasible and efficient.

Overall, there was high agreement on PRECIS-2 scores across types of raters as summarized in Table 2. This level of agreement was achieved even though most team members had not worked with the PRECIS-2 before and there was only minimal training. Our hypothesis that the implementation team members would rate the study design as less pragmatic was partially supported. Non-investigator raters scored the study as less pragmatic on dimensions of recruitment, eligibility, organization, analysis and flexibility- adherence but actually provided slightly more pragmatic ratings for setting, flexibility – delivery, follow up, and outcomes. Overall, the biggest difference in rating between groups was still under 1 full point.

There was good differentiation between PRECIS-2 scores on dimensions we wanted to be more pragmatic vs. more explanatory. In particular, the study design was rated as very pragmatic on setting, eligibility, fidelity-adherence, and analyses, even before the protocol refinement process (scores over 4.38). The outcomes were rated as only moderately pragmatic (average score of 3.75), since although the specific outcomes selected were very patient-centered, the scale and scope of the PROs and the resources required to collect them were seen as burdensome to practices and unlikely to be used for clinical care outside the study context.

Despite the general applicability and usefulness of the PRECIS-2 framework [13], there were two issues that created some confusion. First, multiple levels in the study design and the definition of usual care were at times misunderstood. In many pragmatic studies – especially those with cluster-level randomization and implementation strategies – there are multiple levels of recruitment (e.g., system, practice, staff and patient) and outcomes to consider. When questions arose about how to approach ratings involving multiple levels, for example on recruitment of patients vs. practices, vs. clinical staff, we instructed raters to focus on the patient level. This issue should be ameliorated with the newer PRECIS-2-PS which is focused on the ‘provider delivery level’ rather than patient level design issues [14]. Second, there was some confusion among the team between what is a textbook “pragmatic” study design element (i.e., the same as what happens in usual care) and *ease* of application in participating practices. Many things that are consistent with real-world workflows (e.g., needing to call patients to enroll them in SMAs) are not easy. But this does not make the study non-pragmatic. It may actually make the study *more* pragmatic, because these methods are more similar to those necessary in ‘real world care settings’. It would have been *easier* and less burdensome on participating practices if research staff conducted recruitment, but this would have been less pragmatic.

#### Lessons learned and directions for future research

It was possible after brief training to have staff rate the nine PRECIS-2 dimensions. Raters new to the PRECIS-2 system reported that it was helpful to have examples and to proactively anticipate and address frequently asked questions about ratings. The PRECIS-2 helped the team to be purposeful about where it wanted study be more pragmatic. The PRECIS-2 exercise also helped the research team conceptualize and make adaptations necessary to address COVID-19. It would be of interest to see if other research teams have found PRECIS-2 similarly useful to address research challenges due to COVID-19 and to other rapid or major changes to context [34]. Finally, future research should investigate the extent to which different types of raters continue to evaluate PRECIS-2 dimensions similarly over the life course of a pragmatic trial, as well as whether overall pragmatism ratings increase or decrease.

This research has both strengths and limitations. Strengths include the team science approach, stakeholder engagement, and the relatively large number and types of raters who were involved in rating the study design using the PRECIS-2. The concrete illustrations of how to purposefully make a study more or less pragmatic following initial grant submission and in response to major disruptions such as the COVID-19 pandemic should be of use to other research teams. A caveat is that it may have been more challenging to use PRECIS-2 to rate this study where the comparator was another active SMA intervention rather than usual care. Limitations include a) the study did not involve patients or health system leaders in rating the study design (although they were involved in the protocol refinement process); and b) use of PRECIS-2 was not contrasted with alternative approaches to guide pragmatic design choices.

In conclusion, we encourage greater use of PRECIS-2, and the new PRECIS-2-PS for implementation studies, as well as other design and reporting frameworks (e.g., StaRI) as these implementation science and external validity issues are often neglected. We think that such iterative, multi-stakeholder-engaged protocol refinement processes can a) enhance transparent reporting and help address the scientific replication crisis [1, 2]; and b) provide proactive guidance in the planning stage as well as for later protocol adaptations to address fit to context.

## Supplementary Information

**Additional file 1.**

**Additional file 2.** Invested in Diabetes: Shared Medical Appointment Session Observation Guide.

## Data Availability

The datasets used and/or analysed during the current study are available from the corresponding author on reasonable request.
