# Supplementary material for: Protocol refinement for a diabetes pragmatic trial using the PRECIS-2 framework
Source: BMC Health Serv Res. 2021 Oct 2;21:1039. doi: 10.1186/s12913-021-07084-x (PMC8486627; doi:10.1186/s12913-021-07084-x)
Supplement: Supplementary file 1 — Additional file 1. [file 12913_2021_7084_MOESM1_ESM.docx]

Invested in Diabetes

EHR Data Extraction Guide

Year 1 data: July 1, 2017 - June 30, 2020

This data request is the same as the pilot or test data request you successfully sent us in the third or fourth quarter of 2019. The data includes Patient level data, Encounter level data, and a Medication list. Just as with the pilot or test data you will submit the HIPAA limited data set, which is de-identified data with the exception of date of birth and date of service, from your EHR for end of year 1 of this project.  Your data submission is due July 31, 2020.

This data needs to include the unique identifier that has been created by the shared medical appointment group facilitator at your practice. The crosswalk between identifiable information (names, MRN, etc.) and the new study ID are held at your practice and should not be shared with the Invested in Diabetes research staff. The group facilitator will be responsible for sending us survey and attendance data from patients, which should link up with the unique identifier to the EHR data. Each patient should only have 1 identifier created for this project.

The data will be submitted via CU Denver File Server Egnyte as an Excel document, XML, or comma-delimited text file. (You submitted your pilot data using Egnyte so you already have an account set up. If you need assistance, please reach out to Dennis Gurfinkel, DENNIS.GURFINKEL@CUANSCHUTZ.EDU).

All data elements that begin with an asterisk (*) and are *Italicized*, are optional. We would very much appreciate receiving these optional data elements if you are able to do this.

If possible, if the data field equals null, please leave blank. You do not need to insert N/A, a zero, a dash or other notation to indicate no data for that element.

The tables below define each type of data by category of data that will be required.  The data categories include:

Demographic

Medications

Vital Signs/Lab Results

Visit Data

**Demographic Data:** This will be Patient Level Data, meaning one demographic record per patient per data extraction from July 2020.

| Data Element | Notes/Comments/Concerns |
| --- | --- |
| Date of Birth  mm/dd/yyyy |  |
| Sex at Birth   - Male - Female | <http://www.bphcdata.net/docs/uds_rep_instr.pdf>  pg 34. Table 3A |
| Race   - Asian/Other Pacific Islander - American Indian/ - Alaskan Native - Black/African-American - White - More Than One Race - Unreported/refused to report | <http://www.bphcdata.net/docs/uds_rep_instr.pdf>  pg 32 pg 108 Table 7 |
| Primary Insurance | You can choose to include insurance data as Patient Level data OR as Encounter Level data, whichever is easier for your practice. |
| Secondary Insurance |  |
| Ethnicity   - Hispanic/Latino - Non-Hispanic/Latino - Unreported/refused to report | <http://www.bphcdata.net/docs/uds_rep_instr.pdf>  pg 32, pg 108 Table 7 |
| *Gender Identity***   - *Male* - *Female* - *Transgender Male*   *(Female to Male)*   - *Transgender Female ( Male to Female* - *Other* - *Chose Not to Disclose* | [*http://www.bphcdata.net/docs/uds_rep_instr.pdf*](http://www.bphcdata.net/docs/uds_rep_instr.pdf)  *pg 37. Table 3B*  *Most practices did not populate this field until 2018* |
| *Sexual Orientation***   - *Lesbian or Gay* - *Straight* - *Bisexual* - *Something Else* - *Don’t Know* - *Chose Not to Disclose* | [*http://www.bphcdata.net/docs/uds_rep_instr.pdf*](http://www.bphcdata.net/docs/uds_rep_instr.pdf)  *pg 37. Table 3B*  *Most practices did not populate this field until 2018* |

**Sample Demographic Data Table:**

| study_id | date_of_birth | sex_at_birth | race | ethnicity | Primary_ins | Secondary_ins | gender_identity | Sexual_orientation |
| --- | --- | --- | --- | --- | --- | --- | --- | --- |
| 1 | 00/00/0000 |  |  |  |  |  |  |  |

**Medication Data:** This will be Patient Level Data at day in July 2020 of your data pull.

| Data Element | Data Source | Notes/Comments/Concerns |
| --- | --- | --- |
| PersonID/Study ID | Use constructed ID from person data, created by group facilitator |  |
| Date of Data Pull  mm/dd/yyyy | Date attached to local visit record |  |
| Active Medications | Active/current Medication as of date of data Extraction. Include display medication name generic and/or trade name | Current Medications as of date of data Extraction |
| *Start Date of Medication*** | *Optional*** |  |
| *NDC *** | *Optional*** |  |

**Difficult to provide sample Data Table due to high degree of variability within EHR’s, but it should look similar to this:**

| study_id | date_of_data pull | Medication_Display_Name  ( generic/ trade /or both) | Start_Date | Ok to include any other elements such as NDC sig or dosage if that is easier for your given your EHR |
| --- | --- | --- | --- | --- |

**Lab/Vital Signs Data:** This will be Encounter Level Data. You will include a row of data for each event between start date of July 1, 2017 and June 30, 2020.

| Data Element  Want all results between July 1, 2017 and June 30, 2020 | Data Source | Notes/Comments/Concerns |
| --- | --- | --- |
| PersonID | Unique ID, generated at practice level |  |
| Date of Service | mm/dd/yyyy |  |
| Lab Name and Lab Units | - HbA1c % - Triglyceride mg/dl - HDL mg/dl - LDL mg/dl - Total Cholesterol mg/dl - *GFR*** - *Serum Creatinine mg/dl*** - Systolic BP - Diastolic BP - BMI - *Height*** - *Weight*** | LOINC code or CPT code, 83036  LOINC code or CPT code, 84478  LOINC code or CPT code, 83718  LOINC code or CPT code, 83721  LOINC code or CPT code, 88061, 82465  LOINC code or calculated lab  LOINC code or CPT code, 82565 |
| *Lab LOINC Code* | *Optional*** |  |
| Lab Value/Result | Numeric value associated with lab |  |

**Sample Lab/Vital Signs Data Table: (Can submit in 1 or 2 tables, depending on your preference)**

| study_id | date_of_service | Systolic BP | Diastolic BP | BMI | Height in feet/inches or inches | Weight in pounds |
| --- | --- | --- | --- | --- | --- | --- |

(There will be multiple rows for same patient to capture all vital sign values)

| study_id | date_of_service | HbA1c% | Triglycer mg/dl | HDL mg/dl | LDL mg/dl | Total Cholesterol mg/dl | GFR | Serum Creatinine  mg/dl |
| --- | --- | --- | --- | --- | --- | --- | --- | --- |
| 1 | 07/01/2017 |  |  |  |  |  |  |  |
| 1 | 07/01/2018 |  |  |  |  |  |  |  |

(There will be multiple rows for same patient to capture all labs values)

**OR**

| study_id | date_of_service | Lab Type | Lab Value |
| --- | --- | --- | --- |
| 1 | 07/01/2017 | HbA1c% |  |
| 1 | 07/01/2018 | HDL |  |

**Visit Data:** This will be Encounter Level Data. You will include a row of data for each event between start date of July 1, 2017 and June 30, 2020. (You can exclude dental visits from your data.)

| Data Element | Data Source | Notes/Comments/Concerns |
| --- | --- | --- |
| PersonID/Study ID | Use constructed ID from person data, created by group facilitator |  |
| Date of Service  mm/dd/yyyy | Date attached to local visit record | Use start date if more than one day involved? |
| Visit Type | Send local visit type codes  (Be sure to include group visit type) | Want all outpatient visits |
| Provider Type | Examples:  MD/DO/NP/PA  BHP/Social Worker  RN | Invested will standardize provider types to OMOP list |
| Primary Insurance | Typically in demographic section of EHR | You can choose to include insurance data as Patient Level data OR as Encounter Level data, whichever is easier for your practice. |
| Secondary Insurance | Typically in demographic section of EHR |  |
| DxCode1 | ICD10 Codes noted in each encounter |  |
| DxCode2 | ICD10 Codes noted in each encounter |  |
| DxCode3 | ICD10 Codes noted in each encounter |  |
| DxCode4 | ICD10 Codes noted in each encounter |  |
| DxCode5 | ICD10 Codes noted in each encounter |  |
| *Med Reconciliation*** | *Completed/not completed at visit* |  |
| *Tobacco Status*** | Completed/not completed at visit  *Typically a check box or drop down field in EHR* |  |
| *Tobacco Cessation Counseling*** | Completed/not completed at visit  *CPT code 99406 or 99407*  *eCQM V138.5*  [*https://ecqi.healthit.gov/ecqm/measures/cms138v5*](https://ecqi.healthit.gov/ecqm/measures/cms138v5) |  |
| *Foot Exam*** | Completed/not completed at visit  *CPT? Or Snomed? Depending on EHR*  *eCQM 126.6*  [*https://ecqi.healthit.gov/ecqm/measures/cms123v6*](https://ecqi.healthit.gov/ecqm/measures/cms123v6) |  |
| *Eye Exam*** | Completed/not completed at visit  *CPT? Or Snomed? Depending on EHR*  *eCQM 131.6*  [*https://ecqi.healthit.gov/ecqm/measures/cms131v6*](https://ecqi.healthit.gov/ecqm/measures/cms131v6) | *Excludes Snellen eye chart* |

**Sample Visit Data Table:**

(Primary and Secondary insurance by date of service may or may not be available in your EHR. Often times when insurance is updated, past insurance data is removed)

You can submit ICD10 as second data table or include with the other clinical data components. Whichever is easier for you.

| study_id | date_of_service | Visit_Type | Provider_Type | Payor_primary | Payor_sec | ICD10_1 | ICD10_2 | ICD10_3 | ICD10_4 | ICD10_5 |
| --- | --- | --- | --- | --- | --- | --- | --- | --- | --- | --- |

Table continued:

| Med_Reconciliation | Tobacco_Status | Tobacco_Cessation_Counseling | Foot_ Exam | Eye_Exam |
| --- | --- | --- | --- | --- |
