# Supplementary material for: Protocol refinement for a diabetes pragmatic trial using the PRECIS-2 framework
Source: BMC Health Serv Res. 2021 Oct 2;21:1039. doi: 10.1186/s12913-021-07084-x (PMC8486627; doi:10.1186/s12913-021-07084-x)
Supplement: Supplementary file 2 — Additional file 2. Invested in Diabetes: Shared Medical Appointment Session Observation Guide. [file 12913_2021_7084_MOESM2_ESM.docx]

**Invested in Diabetes: Shared Medical Appointment Session Observation Guide**

Session start time: _________ AM/PM End time: _________ AM/PM Length of session: _________ minutes

Session number for this cohort: ________ (*ask facilitator to indicate session #)*

**Participants:**

Number of patients in attendance: __________ Number of family/friends in attendance: _______

Number of group facilitator(s) present: _______

Roles present (F=facilitator, P=present): *ask facilitator(s) to indicate which of the following apply*

__ Nurse __ Medical Assistant __ Health Educator __ Certified Diabetes Educator

__ Medical Provider (MD/DO/NP/PA) __ Community Health Worker __ Behavioral Health Provider

__ Peer Mentor __ Other (specify): __________________

**TTIM Curriculum Content Covered:**

*For the observed module, check each topic/component as covered and its approximate duration.*

❑ Module 1: Setting the Stage and Introduction to Diabetes

❑ 1.1 Setting the Stage: ❑ < target range (<15 min) ❑ w/in target range (15 min) ❑ > target range (>15

min)

❑ 1.2 Learning about Yourself & Your Starting Point: ❑ < target range (<45 min) ❑ w/in target range (45

min) ❑ > target range (>45 min)

Time spent completing patient surveys: start time: ______________ end time: _________

Survey results discussed: _________________ minutes

❑ 1.3 An Introduction to Diabetes: ❑ < target range (<20) ❑ w/in target range (20 min) ❑ > target range

(>20 min)

❑ 1.4 Diabetes Complications: ❑ < target range (<20) ❑ w/in target range (20 min) ❑ > target range (>20

min)

❑ 1.5 The Benefits of Change & Setting Yourself Up for Success: ❑ < target range (<15 min) ❑ w/in target

Range (15 min) ❑ > target range (>15 min)

❑ 1.6 Patient Selection of Topic Choice: ❑ < target range (<15 min) ❑ w/in target range (15 min) ❑ >

target range (>15 min)

❑ Summary: ❑ < target range (<5) ❑ w/in target range (5 min) ❑ > target range (>5 min)

TTIM visuals/handouts referenced: ❑ 1 ❑ 2 ❑ 3 ❑ 4 ❑ 5

Comments (topics covered out of order, non-TTIM materials used, non-TTIM content covered): _______________________________________________________________________________________________________________________________________________________________________________________________________________________________________________________________

__________________________________________________________________________________________________________________________________________________________________________

❑ Module 2: Diabetes Basics

❑ Home practice review: ❑ < target range (<10 min) ❑ w/in target range (10 min) ❑ > target range (>10 min)

❑ 2.1. Symptoms of High and Low Blood Sugar: ❑ < target range (< 25 min) ❑ w/in target range ❑ > target range (>25)

❑ 2.2. Importance of Glucose Monitoring: ❑ < target range (<15 min) ❑ w/in target range (15 min) ❑ > target range (>15 min)

❑ 2.3. Taking Care of Your Feet: ❑ < target range (<15 min) ❑ w/in target range (15 min) ❑ > target range (>15 min)

❑ 2.4. Staying on Track with Medication Treatments: ❑< target range (<20) ❑ w/in target range (20 min) ❑> target range (>20 min)

❑ 2.5. Personal Goal Setting: ❑ < target range (<30 min) ❑ w/in target range (30 min) ❑ > target range (>30 min)

❑ Summary: ❑ < target range (<5 min) ❑ within target range (5 min) ❑ > target range (>5 min)

❑ Home practice assigned: ❑ 1 ❑ 2

TTIM visuals/handouts referenced: ❑ 1 ❑ 2 ❑ 3 ❑ 4 ❑ 5 ❑ 6 ❑ 7 ❑ 8 ❑ 9

Comments (topics covered out of order, non-TTIM materials used, non-TTIM content covered): _______________________________________________________________________________________________________________________________________________________________________________________________________________________________________________________________

_____________________________________________________________________________________

_____________________________________________________________________________________

❑ Module 3: Problem Solving and Talking to Your Doctor

❑ Home practice review: ❑ < target range (<15 min) ❑ w/in target range (15 min) ❑ > target range (>15 min)

❑ 3.1. Problem-Solving Skills: ❑ < target range (<10 min) ❑ w/in target range (10 min) ❑ > target range (>10 min)

❑ 3.2. The IDEA Approach: ❑ < target range (<30 min) ❑ w/in target range (30 min) ❑ > target range (>30 min)

❑ 3.3 Communication challenges ❑ < target range (<15 min) ❑ w/in target range (15 min) ❑ > target range (>15 min)

❑ 3.4 Tools for Communication with Healthcare Providers: ❑ < target range (<30 min) ❑ w/in target range (30 min) ❑ > target range (>30 min)

❑ 3.5 Personal Care Plan: ❑ < target range (<15 min) ❑ w/in target range (15 min) ❑ > target range (>15 min)

❑ Summary: ❑ < target range (<5 min) ❑ w/in target range (5 min) ❑ > target range (>5 min)

❑ Home practice assigned: ❑ 1 ❑ 2 ❑ 3 ❑ 4

TTIM visuals/handouts referenced: ❑ 1 ❑ 2 ❑ 3 ❑ 4 ❑ 5

Comments (e.g., topics covered out of order, non-TTIM materials used, non-TTIM content covered): __________________________________________________________________________________________________________________________________________________________________________

__________________________________________________________________________________________________________________________________________________________________________

_____________________________________________________________________________________

❑ Module 4A: For All Diabetes Populations: Coping with Stress and Getting the Support You Need

❑ Home practice review: ❑ < target range (<5 min) ❑ w/in target range (5 min) ❑ > target range (>5 min)

❑ 4A.1 Stress and Diabetes: ❑ < target range (<20 min) ❑ w/in target range (20 min) ❑ > target range (>20 min)

❑ 4A.2 Coping with Stress and Emotions: ❑ < target range (<40 min) ❑ w/in target range (40 min) ❑ > target range (>40 min)

❑ 4A.3 Your Support System: ❑ < target range (<25 min) ❑ w/in target range (25 min) ❑ > target range (>25 min)

❑ 4A.4 Talking with Your Supports & the Plus-Minus-Alternative Strategy: ❑< target range (<25 min) ❑ w/in target range ( 25 min) ❑ > target range (>25 min)

❑ Summary: ❑ < target range (5 min) ❑ w/in target range (5 min) ❑ > target range (>5 min)

❑ Home practice assigned: ❑ 1

TTIM visuals/handouts referenced: ❑ 1 ❑ 2 ❑ 3 ❑ 4

Comments (e.g., topics covered out of order, non-TTIM materials used, non-TTIM content covered): _______________________________________________________________________________________________________________________________________________________________________________________________________________________________________________________________

_____________________________________________________________________________________

❑ Module 4B: For SPMI Populations: Coping with stress, mental health conditions, and diabetes

❑ Home practice review: ❑ < target range (<5 min) ❑ within target range (5 min) ❑ > target range (>5 min)

❑ 4B.1 Having a Mental Health Condition & Diabetes: ❑ < target range (< 5 min) ❑ w/in target range (5 min) ❑ > target range (>5 min)

❑ 4B.2 Facts & Myths about Mental Health Conditions: ❑ < target range (<10 min) ❑ w/in target range (10 min) ❑ > target range (>10 min)

❑ 4B.3 Stigma of MHC & Strategies to Cope with Stigma: ❑ < target range (<15 min) ❑ w/in target range (15 min) ❑ > target range (>15 min)

❑ 4B.4 Personal Symptom Profile: ❑ < target range (<15 min) ❑ w/in target range (15 min) ❑ > target (>15 min)

❑ 4B.5 My Stress Action Plan: ❑ < target range (<15 min) ❑ w/in target range (15 min) ❑ > target (>15 min)

❑ 4B.6 Medications & Psychological Treatments for Mental Health: ❑< target range (<10 min) ❑ w/in target (10 min) ❑> target range (>10 min)

❑ 4B.7 Mental Health Treatments and Diabetes: ❑< target range (<10 min) ❑ w/in target (10 min) ❑> target range (>10 min)

❑ 4B.8 Triggers of Relapse: ❑ < target range (<15 min) ❑ w/in target range (15 min) ❑ > target (>15 min)

❑ 4B.9 Substance Use & Its Effects on Mental Health & on Diabetes: ❑ < target range (<15 min) ❑ w/in target range (15 min) ❑ > target (>15 min)

❑ Summary: ❑ < target range (<5 min) ❑ w/in target range (5 min) ❑ > target (>5 min)

❑ Home practice assigned: ❑ 1

TTIM visuals/handouts referenced: ❑ 1 ❑ 2 ❑ 3 ❑ 4 ❑ 5 ❑ 6 ❑ 7 ❑ 8 ❑ 9 ❑ 10 ❑ 11 ❑ 12 ❑ 13

Comments (e.g., topics covered out of order, non-TTIM materials used, non-TTIM content covered):

_________________________________________________________________________________________________________________________________________________________________________________________________________________________________________________________________________________________________________________________________________________________________________________________________________________________________________

❑ Module 5: Nutrition and Healthy Eating

❑ Home practice review: ❑ < target range (<10 min) ❑ w/in target range (10 min) ❑ > target (>10 min)

❑ 5.1 Nutrition for Health: ❑ < target range (<30 min) ❑ w/in target range (30 min) ❑ > target (>30 min)

❑ 5.2 Reading Food Labels: ❑ < target range (<15 min) ❑ w/in target range (15 min) ❑ > target (>15 min)

❑ 5.3 Carbohydrate Counting: ❑ < target range (<20 min) ❑ w/in target range (20 min) ❑ > target (>20 min)

❑ 5.4 Portion Control and the Plate Method: ❑ < target range (<20 min) ❑ w/in target range (20 min) ❑ > target (>20 min)

❑ 5.5 Problem Solving for Healthy Eating: ❑ < target range (<20 min) ❑ w/in target range (20 min) ❑ > target (>20 min)

❑ Summary: ❑ < target range (<5 min) ❑ w/in target range (5 min) ❑ > target (>5 min)

❑ Home practice assigned: ❑ 1 ❑ 2 ❑ 3

TTIM visuals/handouts referenced: ❑ 1 ❑ 2 ❑ 3 ❑ 4 ❑ 5 ❑ 6 ❑ 7 ❑ 8 ❑ 9 ❑ 10 ❑ 11 ❑ 12 ❑ 13 ❑ 14 ❑ 15 ❑ 16

Comments (e.g., topics covered out of order, non-TTIM materials used, non-TTIM content covered): _________________________________________________________________________________________________________________________________________________________________________________________________________________________________________________________________________________________________________________________________________________________________________________________________________________________________________

❑ Module 6: Lifestyle Change – Physical Activity, Sleep, and Good Habits

❑ Home practice review: ❑ < target range (<15 min) ❑ w/in target range (15 min) ❑ > target range (>15 min)

❑ 6.1 Physical Activity Benefits and Recommendations: ❑ < target range (<30 min) ❑ w/in target range (30 min) ❑ > target range (>30 min)

❑ 6.2 Making Physical Activity Part of a Healthy Lifestyle: ❑ < target range (<30 min) ❑ w/in target range (30 min) ❑ > target range (>30 min)

❑ 6.3 Getting Physical Activity in Your Community: ❑ < target range (<15 min) ❑ w/in target range (15 min) ❑ > target range (>15 min)

❑ 6.4 The Importance of a Daily Routine & Good Sleep Habits: ❑ < target range (25 min) ❑ w/in target range (25 min) ❑ > target range (>25 min)

❑ Summary: < target range (<5 min) ❑ w/in target range (5 min) ❑ > target range (>5 min)

❑ Home practice assigned: ❑ 1 ❑ 2

TTIM visuals/handouts referenced: ❑ 1 ❑ 2 ❑ 3 ❑ 4A ❑ 4B ❑ 5 ❑ 6 ❑ 7 ❑ 8 ❑ 9 ❑ 10 ❑ 11 ❑ 12 ❑ 13

Comments (topics covered out of order, non-TTIM materials used, non-TTIM content covered): _________________________________________________________________________________________________________________________________________________________________________________________________________________________________________________________________________________________________________________________________________________________________________________________________________________________________________

❑ Module 7: Reflection and Acknowledgment of Progress

❑ 7.1 Surveys & Reflection upon Group Progress: < target range (<30 min) ❑ w/in target range (30 min) ❑ > target range (>30 min)

Time spent completing patient surveys: start time: ______________ end time: _________

Survey results discussed: _________________ minutes

❑ 7.2 Celebration: ❑ < target range ❑ within target range ❑ > target range

❑ 7.3 Plans for Follow-Up: ❑ < target range ❑ within target range ❑ > target range

TTIM visuals/handouts referenced: ❑ 1 ❑ 2 ❑ 3

**Visits with the Prescribing Provider:**

1. Number of patients stepping out for visits with the prescribing provider *during* session: ________
2. Number of patients visiting with prescribing provider *before* session (ask facilitator): _________
3. Number of patients visiting with prescribing provider *after* session (ask facilitator): __________

**Group Facilitation Style:**

1. Overall, to what extent did the facilitator(s) follow the TTIM manual script?

❑ Read the entire script verbatim

❑ Mostly followed the script verbatim with some paraphrasing

❑ Generally followed the script “in spirit” with substantial paraphrasing

❑ Somewhat followed the script “in spirit” and incorporated own content/concepts

❑ Did not follow the script at all (used own content and concepts entirely)

1. Overall, what was the balance of didactic presentation/lecturing vs group discussion during the session?

❑ *Entirely didactic* presentation/lecturing

❑ *Primarily didactic* with a little group discussion

❑ *Balanced* mix of didactics and group discussion

❑ *Primarily group discussion* with a little didactics

❑ *Entirely group discussion*

1. Overall, to what extent did the facilitator(s) demonstrate effective group facilitation techniques, such as encouraging sharing by all participants, asking open-ended questions, gathering ideas, balancing viewpoints, and keeping the flow of the group moving along productively?

❑ *Poor* group facilitation skills demonstrated

❑ *Fair* group facilitation skills demonstrated

❑ *Good* group facilitation skills demonstrated

❑ *Very good* group facilitation skills demonstrated

❑ *Excellent* group facilitation skills demonstrated

1. Overall, to what extent did the facilitator(s) demonstrate *autonomy support*? *Autonomy support is defined as: “practitioners’ eliciting and acknowledging patients’ perspectives, supporting their initiatives, offering choice about treatment options, and providing relevant information, while minimizing pressure and control.” Being autonomy-supportive may look like: encouraging participants to identify what and how they want to pursue their goals, encouraging people to come up with solutions for their own problems, suggesting a variety of options or choices for resources or strategies (contrast with: demanding, controlling or directing participants to the goals or strategies they believe participants should pursue; telling people what to do or how to solve their problems, offering limited or no choices)*

❑ Very controlling

❑ Somewhat controlling

❑ Equally controlling/autonomy supportive

❑ Somewhat autonomy supportive

❑ Very autonomy supportive

Provide an example of how the facilitator demonstrated autonomy support: __________________________________________________________________________________________________________________________________________________________________________________________________________________________________________________________________________________________________________________________________________________________________________________________________________________________

1. Overall, to what extent did the facilitator(s) demonstrate *effectance* (*competence) support*? *Effectance support is defined as: “practitioners’ providing structure and positive informational feedback.” Being effectance-supportive may look like: praising or encouraging patient goal setting and progress towards achieving goals, providing structure for setting appropriately challenging goals, providing consistent structure and feedback on expectations and benchmarks for success (contrast with: encouraging overly challenging goals, being inconsistent in expectations, criticizing or otherwise discouraging progress)*

❑ Very discouraging

❑ Somewhat discouraging

❑ Equally discouraging/effectance supporting

❑ Somewhat effectance supporting

❑ Very effectance supporting

Provide an example of how the facilitator(s) demonstrated effectance support: __________________________________________________________________________________________________________________________________________________________________________________________________________________________________________________________________________________________________________________________________________________________________________________________________________________________

1. Overall, to what extent did the facilitator(s) demonstrate *relatedness support*? *Relatedness support is defined as: “practitioners’ demonstrating caring for the individual person.” Being relationally supportive may look like: asking about or eliciting patients’ personal experiences, acknowledging or praising sharing of personal preferences, explaining or providing examples in terms consistent with patients’ personal experience or preferences (contrast with: being impersonal or rejecting of the individual)*

❑ Very impersonal

❑ Somewhat impersonal

❑ Equally impersonal/relationally supportive

❑ Somewhat relationally supportive

❑ Very relationally supportive

Provide an example of how the facilitator demonstrated relatedness support: ____________________________________________________________________________________________________________________________________________________________________

______________________________________________________________________________________________________________________________________________________________________________________________________________________________________________________

**Group Participation and Dynamics:**

1. To what extent did participants contribute to the group discussion?

❑ The group was dominated by one or two people

❑ Most participants had an opportunity to contribute but at least one did not contribute

❑ All participants had an opportunity to contribute

1. What was the tone of group discussion?

❑ Excited/encouraged ❑ Subdued ❑ Dismissive/Hostile ❑ Other: ___________

1. What was the sense of camaraderie among participants?

❑ Strong ❑ Somewhat ❑ Weak

1. If a peer mentor was present, to what extent did they contribute to the group discussion, such as by sharing personal experiences, providing encouragement, or suggesting resources?

❑ A lot ❑ A moderate amount ❑ Very little ❑ N/A (no peer mentor present)

Any other comments/observations about the session?

________________________________________________________________________________________________________________________________________________________________________________________________________________________________________________________________________________________________________________________________________

____________________________________________________________________________________________________________________________________________________________________
